# Potentials 'R'Us web-server for protein energy estimations with coarse-grained knowledge-based potentials

**DOI:** 10.1186/1471-2105-11-92

**Published:** 2010-02-17

**Authors:** Yaping Feng, Andrzej Kloczkowski, Robert L Jernigan

**Affiliations:** 1 Department of Biochemistry, Biophysics, and Molecular Biology, Iowa State University, Ames, IA 50011-0320, USA; 2 LH Baker Center for Bioinformatics and Biological Statistics, Iowa State University, Ames IA 50011-3020, USA

## Abstract

**Background:**

Knowledge-based potentials have been widely used in the last 20 years for fold recognition, protein structure prediction from amino acid sequence, ligand binding, protein design, and many other purposes. However generally these are not readily accessible online.

**Results:**

Our new knowledge-based potential server makes available many of these potentials for easy use to automatically compute the energies of protein structures or models supplied. Our web server for protein energy estimation uses four-body potentials, short-range potentials, and 23 different two-body potentials. Users can select potentials according to their needs and preferences. Files containing the coordinates of protein atoms in the PDB format can be uploaded as input. The results will be returned to the user's email address.

**Conclusions:**

Our Potentials 'R'Us server is an easily accessible, freely available tool with a web interface that collects all existing and future protein coarse-grained potentials and computes energies of multiple structural models.

## Background

Prediction of protein three-dimensional structures from their amino acid sequences is one of the important goals of computational biology. The rate of determination of protein structures by experimental methods such as nuclear magnetic resonance (NMR) spectroscopy and X-ray crystallography cannot, unfortunately, catch up with the extremely rapid growth of protein sequences from the mass-scale genome sequencing studies. Additionally experimental structure determination methods are quite expensive both in terms of equipment and human effort, mostly because of difficulties in obtaining high quality protein crystals [1]. Because of this, the computational prediction of protein structure from amino acid sequence becomes increasingly important.

There are two types of computational strategies for predicting protein structure [2]: template-based protein structure modeling and *ab initio *structure prediction. *Ab initio *methods try to build three-dimensional protein models "from scratch", and are based on physical considerations rather than on the use of a previously solved individual structure. *Ab initio *procedures require significant computational resources to perform searches throughout the whole conformational space to seek the lowest energy conformers, and therefore are applicable only for relatively small proteins. Template-based protein modeling utilizes known protein structures as the starting points for structure prediction. These methods may also be divided into two categories: comparative (or homology) modeling and protein threading (fold recognition). Homology modeling is based on the assumption that two homologous proteins have similar structures. When the query sequence has sequence identity of approximately 30% or higher in comparison with a sequence having a known structure available from the Protein Data Bank (PDB), we use homology modeling to predict protein structure. When only distant homologs with low sequence identity to the query sequence can be found in PDB, we use protein threading algorithm to select a protein fold. The basic idea of protein threading is that the target sequence for which the structure is being predicted is threaded through the backbones of a collection of template protein structures and energy scores are calculated for each sequence-structure alignment using knowledge-based coarse-grained potentials such as four-body potentials [3], two-body potentials [4,5] and short-range potentials [6] employed in this server.

All two-body potentials available on our server were previously analyzed in 2005 by Pokarowski *et al. *[7], who compared 29 different two-body potentials currently used in computational biology, and approximated them with simple functions of the physical properties of amino acids. All these pair-contact potentials can be expressed as symmetric matrices. The best known representatives of the two-body potentials, are the MJ potentials, which were first introduced by Miyazawa and Jernigan in 1985 [4], and then rederived using an updated, larger protein dataset in 1996 [5]. Both papers are highly cited, according to the ISI Web of Science. MJ potentials were derived from the statistics of inter-residue contacts occurring in a set of proteins using the quasi-chemical approximation with an approximate estimation of the chain connectivity effects [4,5]. There are two kinds of MJ two-body contact potentials, e_ij _and e_ij'_, denoted as MJ1-3 and MJ1h-3h in our web server. The later one (marked with the suffix "h") includes the energy of transfer of amino acids from water to the protein environment. The pair-contact energies e_ij _and e_ij' _(in *k*_*B*_*T *units, where *k*_*B *_is the Boltzmann constant and *T *is temperature) were derived based on the assumptions that  and . Here indices *i*, *j*, and 0 represent residue *i*, residue *j *and solvent respectively, and  is the statistical average of the number of contacts *n*_*ij *_between residues of types *i *and *j*. More details of the derivation of MJ two-body contact potentials are given in reference [4]. Since the correlation coefficients between MJ2h and MJ1h and between MJ3 and MJ2, as shown in reference [7] are quite high, we only use MJ2h and MJ3 for threading purposes in our potential server. More details about all of the two-body potentials and their abbreviations used in our server can be found in Pokarowski's paper [7]. The four-body contact potentials and short-range interaction potentials have been derived by considering different aspects of protein structures than those used to derive pair-contact potentials. The four-body contact potentials [3] are appropriate for representing the cooperative parts of the protein folding process, and we have shown that they are quite successful for recognizing the native structures among hundreds or even thousands of decoys from the Decoys'R'Us database [8]. Short-range interaction energies allow us to estimate free energies from the statistical distribution of local conformational descriptors [6]. We usually assume that the lower the computed energy score; the better is the predicted structure in accordance with the thermodynamic hypothesis that the native state of a protein has the lowest free energy.

Potential energies are essential for all protein structure prediction methods, and can be used either to guide the conformational search process, or to select a native structure from a preselected set of possible models of the structure. Protein contact potentials are also used in protein design, protein docking, simulations of folding, and in many other applications. Knowledge-based optimization potentials are usually derived from the known protein structures solved by X-ray crystallography or NMR, by fitting their values to optimize the recognition of the native structures from sets of computer generated structures (decoys) [9]. In contrast to atomic potentials based on real physical interactions, knowledge-based potentials incorporate and average over many different physical interactions, such as hydrophobic, electrostatic, hydrogen-bond and cation-π interactions, and so these statistically derived potentials do not necessarily reflect true energies but rather are effective ones averaged over many of the atomic details. Results of the Critical Assessment of Techniques for Protein Structure Prediction (CASP) show that the groups using knowledge-based statistical potentials have been more successful for both *ab initio *structure prediction and template based modeling [10-15].

Although most of the potentials available on our server were derived a number of years ago, these potentials have never been collected nor made accessible to the public through a web server. Our knowledge-based potential server will overcome this deficiency and should be an extremely convenient location for any researcher to compute and compare energies of different protein conformations of the same protein.

## Implementation

**Figure 1:**
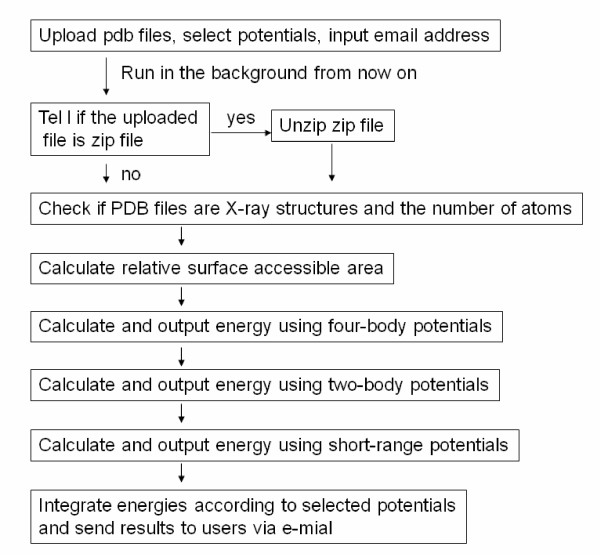
**The flowchart for the potential server**.

Figure 1 shows schematically how our potential server works. Our potential server computes energies for the supplied set of protein conformations. However it will not generate conformations for the submitted amino acid sequence.

All 27 different knowledge-based potentials included in our server are listed on the top of the web page at http://gor.bb.iastate.edu/potential. The abbreviations of the potentials employed in our server are the same as in reference [7]. Details of each of the knowledge-based potentials and the related literature references can be accessed by clicking the corresponding name on the list of 27 potentials. All of these 27 different potentials except the general four-body potentials have been published in the literature over the last 25 years. The general four-body potentials (the second entry on the list of the potentials) are the newest (not yet published) modifications of our recently published four-body contact potentials [3] that are also included in the server (see the first entry on the list). We will update the literature referring to the general four-body potentials after the publication of the relevant paper. We should also note that our potential server is an ongoing project; the list of potentials included is neither final nor complete, and we will add more knowledge-based potentials (especially those uncorrelated with presently included ones) that have either been omitted now or that will be published in the future. We are open to comments and suggestions for including new knowledge-based potentials and for improvement of our web server.

## Results

**Figure 2:**
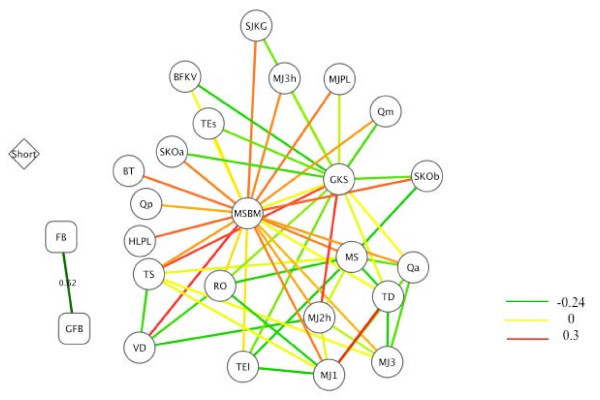
**The correlations among various potentials**. The circle nodes represent all pair-wise potentials, squares four-body potentials, and the diamond the short-range potentials used in this server. The edges represent the Pearson correlations between pairs of potentials. FB denotes four-body potentials, GFB general four-body potentials, and "Short" short-range conformational potentials. (See reference [7] for identities.)

The Pearson correlation between four-body potentials and general four-body potentials is 0.62 (Figure 2). Since the short-range potentials and four-body potentials were derived in a completely different way compared from pairwise potentials, we can not calculate the Pearson correlation for them. Because the highly correlated pairwise potentials may lead to redundant threading results, we show that there are pairwise potentials with low correlations below 0.3 (see Figure 2). The correlation between VD and MSBM is -0.24, the lowest correlation. The users may choose the most different potentials according to Figure 2.

The potential server currently accepts only one file or a set of files corresponding to different conformations of the same protein in the PDB format. The users should compress multiple PDB files into a single zip archive before submitting them to the server. Each PDB file should contain only a single conformation. The server will check whether the uploaded PDB files correspond to NMR or X-ray structures and will check the total number of atoms in a single PDB file. The server will not compute energies either for NMR structures or for PDB files having more than 25000 atoms, and will only send warning messages via email. If the uploaded zip file contains both NMR and X-ray structures, the server can recognize X-ray structures and will compute only energies for all qualified structures. If users want to estimate the energies of NMR structures, they should split the multiple models into separate PDB files before submitting them to the server. We allow the main program to run in the background, so users can close their web browsers once they finish uploading the files. The server first calculates the residue accessible surface area using NACCESS [16] that is used for energy calculations with four-body contact potentials, and then it computes and outputs the energies for the four-body potentials, the two-body potentials and the short-range conformational potentials. Finally the server integrates all of these results into one text output file and sends this file to the email address supplied by the user.

All the structural data files in the PDB format are analyzed on a coarse grained level as follows: the geometric center of all side-chain heavy atoms from one residue is calculated to represent this residue, or if the PDB file has only backbone atoms, then the Cα atom is used to represent the residue.

We have provided one example for users on the website to help them learn how to use the server. This example shows the practical application of our potential server for fold recognition. The supplied zip archive contains 25 PDB files including 1ctf.pdb - the native crystal structure of the C-terminal domain of the ribosomal protein L7/L12 from *Escherichia coli *at a resolution of 1.7 Å [17] and 24 other PDB files that are computer generated conformations, or so called decoys, of 1ctf. The results returned by the server via e-mail show that the native structure has the energy lower than any other decoy when threaded for all potentials except TS, MJ1, and MSBM. The possibility to compute the energies of threadings by using a variety of knowledge-based potentials increases the reliability of fold recognition and may be used in the future to develop improved consensus predictions.

For the above example containing 25 PDB files, it takes the sever about 10 seconds to complete the calculations. We have also tested the potential server on a much larger set of 1783 PDB files for a protein composed of 101 amino acids; in this case it took the server around 9 minutes to compute the results and return them by e-mail. Recently, we have tested the server using 2278 PDB files submitted simultaneously; and it took the server around 15 minutes to return the results by email. The size of the zip file for those 2278 files was 47.1 MB. This shows that the server has the ability to compute a large number of pdb files at one time. It should be convenient for users requiring energies to be calculated for large numbers of computer generated conformations.

The server consists of a Linux box with RedHat Enterprise 3.0 operating system with 4.5 GB RAM and 140 GB hard disk storage. The program code was written in C++ and the web interface has been developed using a CGI script written in HTML and PERL. We may make further improvements in the future to our server by upgrading its hardware and software for enhanced performance depending on the extent of users' demands. Users are encouraged to contact the system administrator via the e-mail provided on the web page to solve any possible problems or to suggest improvements to the functionality and performance of the server.

## Conclusion

The knowledge-based potential server is an easily accessible, freely available tool with a web interface that collects all existing and future protein coarse-grained potentials and computes energies of multiple structural models. It allows evaluation of energies of different protein folds for non-computational biology specialists, and significantly improves the access to a wide variety of knowledge-based potentials. The server accepts multiple structural files in the PDB format (including hundreds or even thousands of decoys) and the results are sent back to users promptly at the supplied e-mail address.

## Availability and requirements

Project home page: http://gor.bb.iastate.edu/potential

Operating system: RedHat Enterprise 3.0 operating system with 4.5 GB RAM and 140 GB hard disk storage

Programming language: C++, Perl, CGI script

License: GNU GPL

The potential server is freely accessible to all users.

## Abbreviations

PDB: Protein Data Bank; NMR: nuclear magnetic resonance; CASP: Critical Assessment of Techniques for Protein Structure Prediction; For all abbreviations for two-body potentials: see reference [7]

## Authors' contributions

YPF, AK and RLJ designed this potential server. YPF implemented this server and wrote the paper. AK and RLJ revised the paper. All authors read and approved the final manuscript.
